# Socioeconomic position across life and body composition in early old age: findings from a British birth cohort study

**DOI:** 10.1136/jech-2013-203373

**Published:** 2014-02-24

**Authors:** David Bann, Rachel Cooper, Andrew K Wills, Judith Adams, Diana Kuh, on behalf of the NSHD scientific and data collection team

**Affiliations:** 1 MRC Unit for Lifelong Health and Ageing at UCL, London, UK; 2 School of Clinical Sciences, University of Bristol, Bristol, UK; 3 Central Manchester University Hospital NHS Foundation Trust, Manchester, UK

**Keywords:** socioeconomic factors, body composition, obesity, body fat distribution, muscle mass

## Abstract

**Background:**

Previous studies have reported associations between lower lifetime socioeconomic position (SEP) and higher body mass index in adulthood, but few have examined associations with direct measures of fat and lean mass which are likely to have independent roles in health and physical functioning.

**Methods:**

We examined associations of SEP across life with dual-energy X-ray absorptiometry measures of fat and lean mass at 60–64 years using data from a total of 1558 men and women participating in the Medical Research Council (MRC) National Survey of Health and Development. We also examined whether associations of childhood SEP with fat and lean mass were explained by preadulthood weight gain (birth weight, 0–7 and 7–20 years) and adult SEP.

**Results:**

Lower SEP across life was associated with higher fat mass and higher android to gynoid fat mass ratio. For example, the mean difference in fat mass index comparing the lowest with the highest paternal occupational class at 4 years (slope index of inequality) was 1.04 kg/m^1.2^ in men (95% CI 0.09 to 1.99) and 2.61 in women (1.34 to 3.89), equivalent to a 8.6% and 16.1% difference, respectively. After adjustment for fat mass, lower SEP across life was associated with lower lean mass in women, while only contemporaneous household income was associated in men. Associations between childhood SEP and outcomes were partly explained by preadulthood weight gain and adult SEP.

**Conclusions:**

This study identified lifetime socioeconomic patterning of fat and lean mass in early old age. This is likely to have important implications and may partly explain socioeconomic inequalities in health and physical functioning.

## Introduction

Systematic reviews have shown that lower socioeconomic position (SEP) in both childhood and adulthood is associated with increased adult obesity risk (ie, high body mass index (BMI)), with associations in women typically stronger and more consistent across adult SEP indicators than in men.^1^ ^2^ Given the deleterious consequences of obesity, these associations have important implications and may partly explain observed socioeconomic inequalities in health and physical functioning.^3^ ^4^ However, fewer studies have examined associations between SEP and direct measures of fat and lean (muscle) mass which BMI does not distinguish; lean mass may also be socioeconomically patterned, and is likely to have independent roles in health and functioning.^5^ Low lean mass has been related to worse physical functioning,^6^ lower bone mineral content,^7^ adverse glucose metabolism,^8^ lower basal metabolic rate^9^ and is also an essential component of consensus definitions of sarcopenia, a geriatric syndrome of increasing public health concern.^10^

Previous studies that examined associations between SEP and direct measures of fat and/or lean mass have produced inconsistent findings.^11–19^ In most of these studies, SEP was not the main explanatory factor investigated and the magnitude of associations were therefore not presented. Also, these have typically used single indicators of SEP ascertained at one point in life, limiting the understanding of how SEP at different life stages affects subsequent body composition. Previous studies have not considered whether SEP is associated with lean mass independently of fat mass. Due to adaptive mechanisms, gains or losses in fat mass that take place in early to mid-adulthood typically lead to respective gains or losses in lean mass;^20^ ^21^ as such, associations with fat mass may drive and thereby confound associations with lean mass.

**Figure 1:**
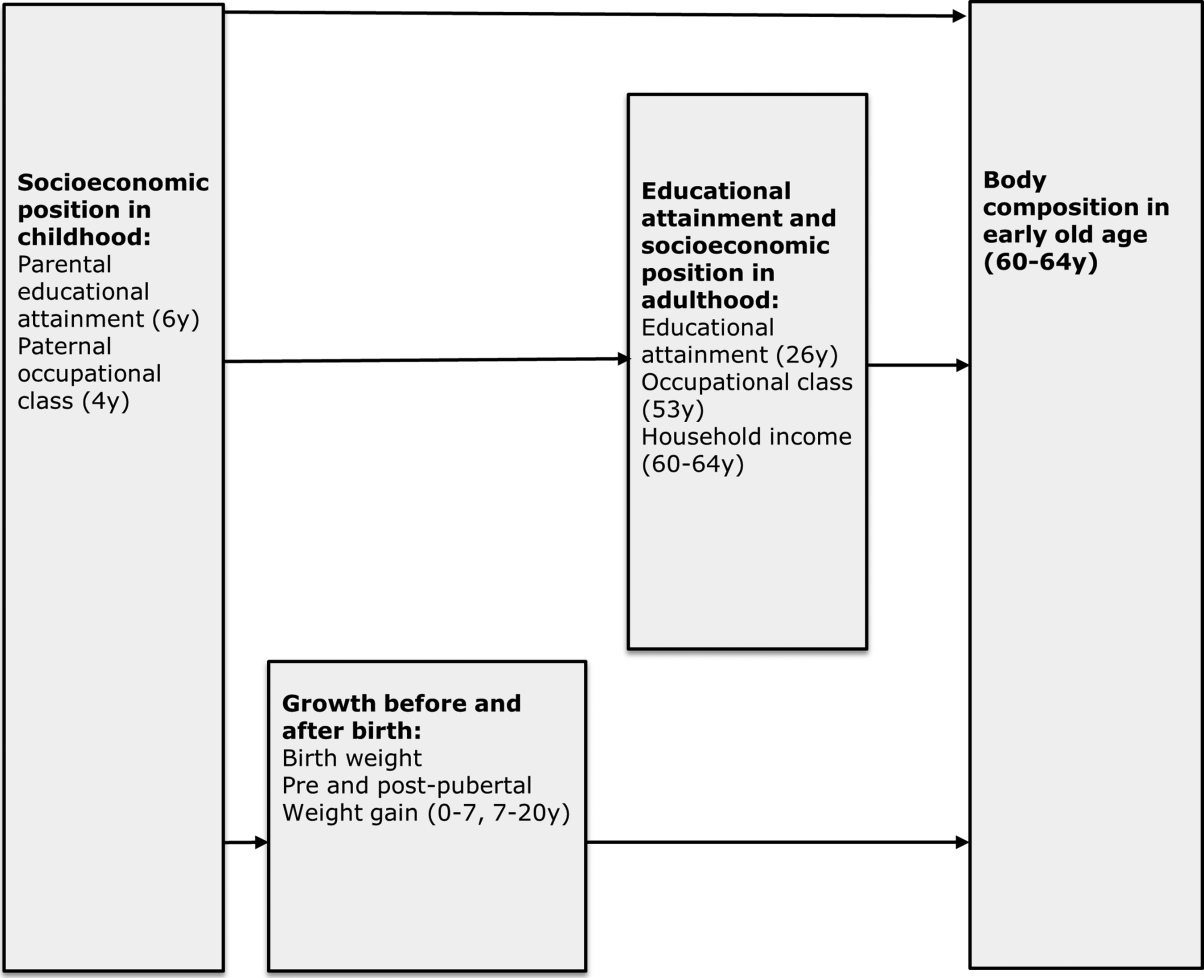
Conceptual framework illustrating how socioeconomic position in childhood may affect adult body composition.

The objectives of this study were to examine the associations of SEP across life with direct measures of fat and lean mass in early old age using data from a British birth cohort study. We hypothesised that lower SEP across life would be associated with higher fat and lower lean mass due to the socioeconomic patterning of the determinants of these masses. Consistent with previous studies using BMI as an outcome,^1^ ^2^ we expected that SEP differences in fat mass would be stronger in women than men. This may be due to the stronger socioeconomic patterning of fat mass determinants among women and/or due to these determinants having a greater impact on women than men: for both biological (sex) and social/cultural (gender) reasons, women may be more susceptible to an obesogenic environment than men.^22^ ^23^ We expected that associations with childhood SEP would be independent of adult SEP and partly explained by socioeconomic differences in birth weight and preadulthood weight gain: lower childhood SEP has been associated with lower birth weight,^4^ ^24^ which is related to lower adult lean mass^11^ ^25^ ^26^ and greater weight gain in later childhood and adolescence,^27^ which is related to higher adult fat mass.^11^ ^26^ A visual representation of these relationships is shown in figure 1.

## Methods

### Study sample

The MRC National Survey of Health and Development (NSHD) is a socially stratified sample of 5362 singleton births that took place in 1 week of March 1946 in mainland Britain,^28^ with regular follow-up across life. At 60–64 years, 2856 eligible study members were invited for an assessment at one of six clinical research facilities (CRFs) or a home visit. Invitations were not sent to those who had died (n=778), who were living abroad (n=570), had previously withdrawn from the study (n=594) or who had been lost to follow-up (n=564). Of those invited, 2229 were assessed: 1690 attended a CRF and the remaining 539 were seen at home.^29^ The study received Multi-Centre Research Ethics Committee approval and informed consent was provided by participants.

### Body composition measurement

During the visits to the CRF, measures of body composition were obtained in the supine position using a QDR 4500 Discovery DXA scanner (Hologic Inc, Bedford, Massachusetts, USA) with APEX V.3.1 software as previously described.^26^ From these scans, measures of fat (whole body, android and gynoid) and appendicular (limb) lean mass were obtained and converted into kilograms. The ratio of android to gynoid fat mass was derived (higher values indicating greater fat distribution in the abdomen than hips) and multiplied by 100. Lean mass was defined as mass excluding fat and bone mass, and in all measures data from the head were excluded due to the high proportion of bone mass known to affect the accuracy of soft-tissue measures.^30^ Data on these outcomes were available for 1558 participants.

### SEP across life

Indicators of SEP were selected a priori to capture SEP across life. Indicators of childhood SEP chosen were paternal occupational class at 4 years (the Registrar General's classification (RGSC) modelled in six standard categories) and maternal and paternal educational attainment at 6 years. Maternal and paternal education was classified as follows: (1) primary only, (2) primary and further education (no qualifications obtained), (3) secondary only (or primary and further education) and (4) secondary and further education or higher. Highest educational level achieved by 26 years was classified using the Burnham scale:^31^ (1) no qualifications, (2) sub-GCE or sub-Burnham C, (3) GCE O level or Burnam C, (4) GCE A level or Burnam B and (5) degree or higher.^31^ Indicators of adult SEP chosen were highest household occupational class at 53 years (RGSC) and self-reported annual household income at 60–64 years (post-tax from all sources in 13 bands from per year: £≤6000 to ≥ 30 000).

### Potential mediators: birth weight and preadulthood weight gain

Birth weight was extracted from birth records a few days after birth, and weight was measured at age 7 and self-reported at age 20. Prepubertal weight gain was calculated from 0 to 7 years and pubertal weight gain from 7 to 20 years.

### Analytical strategy

Height-adjusted indices for fat and appendicular lean mass were created to account for the fact that taller individuals tend to have more fat and lean mass, and account for SEP differences in height. These were created by dividing fat or lean mass (kg) by height (m)^X^ (X=1.2 for fat and 2 for lean mass), where X was calculated so that the resulting index was not correlated with height.^32^

To provide crude estimates of associations, the outcome means were presented by SEP categories and associations examined using linear regression. Slope indices of inequality (SII) were then used to facilitate comparisons of the associations of different SEP indicators,^33^ which account for differences in the distribution of participants across categories of different indicators of SEP. Each indicator was converted into sex-specific ridit scores; individuals in each category were assigned a value equivalent to the proportion of the population with higher SEP than the midpoint of that category. These scores were then each regressed against each outcome with coefficients representing the absolute difference in mean levels of outcome when comparing those with the hypothetically lowest (0) with those of hypothetically highest SEP (1).^33^ Each model was restricted to those with valid data for the SEP indicator used and all body composition outcomes.

All analyses were conducted separately in each sex and sex differences formally tested by including an interaction term. Deviation from linearity was examined using likelihood ratio tests comparing models with each SEP indicator included as a linear and categorical term. As changes in fat mass that take place in early to mid-adulthood typically lead to respective changes in lean mass,^20^ ^21^ associations with fat mass may drive and thereby confound associations with lean mass. Models using appendicular lean mass index as an outcome were therefore additionally adjusted for fat mass index.

To examine whether associations between childhood SEP and outcomes were explained by preadulthood weight gain or adult SEP, associations found in unadjusted analyses (at the p<0.05 level) were further adjusted for birth weight, conditional weight gain from 0 to 7 and 7 to 20 years (with weight at birth and 7 years, respectively, also included in models), and indicators of adult SEP (ie, own educational attainment, occupational class and household income). These analyses were restricted to those with valid data for all SEP indicators, potential mediators and all outcomes.

## Results

### Associations of SEP with fat mass and android to gynoid ratio

The distribution of participants and mean outcomes across categories of each indicator of SEP are shown in table 1. In both sexes, lower SEP in childhood and adulthood were associated with higher fat mass and higher android to gynoid ratio. Table 2 shows these associations with SEP indicators modelled using the SII. The effect sizes differed by SEP indicator: for men, lower own education was most strongly associated with higher fat mass, with a 1.65 (95% CI 0.69 to 2.60) higher fat mass index (kg/m^1.2^) in the lowest compared with highest educational level. In women, paternal educational attainment was most strongly associated, with a 3.67 (2.28 to 5.07) higher fat mass index in the lowest compared with highest paternal educational level. The associations of lower childhood SEP and occupational class in adulthood with higher fat mass were stronger in women than men (table 2A), while associations between lower SEP and higher android to gynoid ratio were similar for most indicators of SEP in both sexes except for own education, where the association was stronger in men (table 2B).

**Table 1:** Mean fat mass, android to gynoid ratio and lean mass levels at age 60–64 years by sex and socioeconomic position across life

	N (M/F)	Fat mass index (kg/m^1.2^) Mean (SD)		Android to gynoid ratio Mean (SD)		Appendicular lean mass index (kg/m^2^) Mean (SD)	
		Men	Women	Men	Women	Men	Women
Sex	1558 (746/812)	12.13 (3.62)	16.23 (5.11)	65.69 (15.35)	44.74 (12.36)	8.01 (0.95)	6.16 (0.87)
*p Value (t test)*		<0.001		<0.001		<0.001	
*Socioeconomic position*							
Paternal occupational class (4 years)							
I—Professional	113 (55/58)	11.6 (3.6)	15.0 (5.0)	59.4 (14.9)	43.4 (12.7)	7.9 (1.0)	6.2 (0.8)
II—Intermediate	282 (133/149)	11.5 (3.8)	15.8 (5.4)	62.1 (16.8)	41.9 (11.3)	7.9 (1.0)	6.2 (0.9)
III—Skilled (non-manual)	334 (157/177)	11.9 (3.4)	15.3 (4.7)	65.6 (15.5)	44.3 (13.2)	8.0 (0.9)	6.0 (0.9)
III—Skilled (manual)	406 (195/211)	13.0 (3.8)	16.7 (5.0)	68.9 (14.5)	45.8 (11.2)	8.1 (1.0)	6.2 (0.9)
IV—Partly skilled	267 (132/135)	12.3 (3.4)	17.3 (5.4)	67.6 (13.9)	47.0 (12.3)	8.1 (0.9)	6.2 (0.9)
V—Unskilled	75 (38/37)	11.3 (3.6)	17.4 (4.6)	64.0 (15.8)	46.7 (13.2)	8.1 (1.1)	6.3 (0.8)
*p Value (trend)*		0.05	<0.001	<0.001	<0.001	0.01	0.73
Maternal educational attainment (6 years)							
Secondary and FE	214 (99/115)	11.8 (3.8)	15.0 (5.2)	61.0 (14.6)	41.6 (12.1)	8.0 (1.0)	6.1 (0.9)
Secondary only	184 (89/95)	11.5 (3.2)	15.6 (4.6)	62.7 (15.1)	43.6 (11.6)	7.9 (1.0)	6.1 (0.8)
Primary and FE	223 (121/102)	12.2 (3.9)	16.1 (5.7)	67.8 (16.6)	44.0 (12.6)	8.0 (0.9)	6.2 (0.8)
Primary only	768 (359/409)	12.5 (3.7)	16.8 (5.1)	67.4 (14.9)	45.9 (12.0)	8.1 (0.9)	6.2 (0.9)
*p Value (trend)*		0.02	<0.001	<0.001	<0.001	0.07	0.41
Paternal educational attainment (6 years)							
Secondary and FE	275 (131/144)	12.1 (3.6)	14.5 (4.3)	62.5 (15.4)	40.2 (11.0)	7.9 (0.9)	6.1 (0.7)
Secondary only	213 (98/115)	11.7 (3.7)	15.9 (5.1)	63.7 (15.1)	43.8 (12.0)	8.1 (1.0)	6.2 (0.9)
Primary and FE	209 (117/92)	11.8 (3.1)	16.1 (5.5)	65.8 (16.1)	43.8 (13.0)	7.9 (0.9)	6.1 (0.9)
Primary only	681 (314/367)	12.6 (3.9)	17.1 (5.2)	68.1 (15.2)	47.0 (12.1)	8.1 (0.9)	6.2 (0.9)
*p Value (trend)*		0.08	<0.001	<0.001	<0.001	0.08	0.19
Own educational attainment (26 years)							
Degree or higher	193 (132/61)	11.6 (3.4)	15.3 (5.2)	62.8 (16.3)	42.0 (12.2)	7.9 (0.9)	6.3 (0.9)
GCE A level or equivalents	472 (232/240)	11.8 (3.5)	15.7 (4.9)	63.6 (14.6)	44.0 (12.5)	8.0 (1.0)	6.1 (0.8)
GCE O level or equivalents	326 (108/218)	12.1 (3.9)	16.2 (5.2)	66.5 (15.2)	45.7 (12.6)	8.0 (0.9)	6.1 (0.9)
Sub-GCE O level or equivalents	110 (40/70)	12.3 (3.3)	16.5 (4.9)	67.4 (17.2)	45.2 (12.2)	7.6 (0.9)	6.1 (0.9)
No qualifications	374 (192/182)	12.9 (3.8)	17.1 (4.9)	68.8 (14.2)	45.7 (12.1)	8.2 (0.9)	6.2 (0.9)
*p Value (trend)*		<0.001	<0.001	<0.001	0.05	0.01	0.86
Occupational class (53 years)							
I—Professional	200 (114/86)	11.6 (3.2)	14.7 (4.2)	63.9 (16.4)	41.3 (11.4)	8.0 (0.9)	6.1 (0.8)
II—Intermediate	800 (376/424)	12.2 (3.6)	16.3 (5.3)	65.6 (15.1)	44.8 (12.2)	8.0 (1.0)	6.2 (0.9)
III—Skilled (non-manual)	325 (139/186)	12.1 (3.6)	16.2 (4.7)	66.0 (15.0)	44.3 (13.4)	8.0 (0.9)	6.0 (0.8)
III—Skilled (manual)	136 (83/53)	12.8 (4.3)	18.1 (6.1)	69.0 (15.0)	47.4 (10.9)	8.2 (0.9)	6.4 (0.9)
IV—Partly skilled	56 (22/34)	11.4 (3.7)	17.7 (4.6)	60.3 (13.5)	50.8 (10.1)	7.9 (1.1)	6.3 (0.9)
V—Unskilled	11 (5/6)	12.0 (2.5)	14.9 (4.7)	84.6 (12.9)	45.3 (20.2)	8.6 (0.9)	5.7 (1.2)
*p Value (trend)*		0.23	<0.001	0.06	<0.001	0.20	0.73
Household income (60–64 years)							
1 (highest)	525 (292/233)	11.7 (3.2)	15.6 (4.6)	64.6 (15.7)	43.8 (12.5)	8.0 (0.9)	6.1 (0.8)
2	372 (172/200)	12.0 (3.5)	15.9 (4.7)	65.1 (14.3)	44.6 (12.4)	8.1 (0.9)	6.1 (0.9)
3	382 (171/211)	12.5 (3.8)	17.0 (5.8)	66.4 (14.6)	45.1 (12.6)	8.0 (1.0)	6.2 (0.9)
4 (lowest)	202 (76/126)	12.6 (4.2)	17.0 (5.3)	68.6 (17.1)	46.4 (12.1)	7.9 (0.9)	6.1 (0.9)
*p Value (trend)*		0.01	<0.001	0.04	0.06	0.72	0.51

Maternal and paternal education classified as follows: (1) primary only, (2) primary and further education (FE) (no qualifications obtained), (3) secondary only (or primary and FE) and (4) secondary and FE or higher. Own education was classified using the Burnham scale:^31^ (1) no qualifications, (2) sub-GCE or sub-Burnham C, (3) GCE O level or Burnam C, (4) GCE A level or Burnam B and (5) degree or higher. Occupational class was that of the highest in the household and derived using the Registrar General's social classification; annual household income was categorised into four groups to aid presentation (per year: £≤6000–11 999, 12 000–20 999, 21 000–29 999, ≥30 000); analyses were restricted to those with valid measures for all body composition outcomes.

**Table 2:** Differences in fat mass, android to gynoid ratio and lean mass (95% CI) at age 60–64 years between the hypothetical lowest and highest socioeconomic position (slope index of inequality)

		(A) Fat mass index (kg/m^1.2^)					(B) Android to gynoid ratio				
	N (M/F)	Men	p Value	Women	p Value	p Value#	Men	p Value	Women	p Value	p Value#
Paternal occupational class (4 years)	710/767	1.04 (0.09 to 1.99)*	0.03	2.61 (1.34 to 3.89)	<0.01	0.06	8.03 (4.06 to 12.01)*	<0.01	5.91 (2.87 to 8.95)	<0.01	0.40
Maternal education (6 years)	668/721	1.27 (0.22 to 2.33)	0.02	2.72 (1.28 to 4.16)	<0.01	0.11	8.22 (3.82 to 12.63)	<0.01	6.12 (2.73 to 9.52)	<0.01	0.46
Paternal education (6 years)	660/718	1.07 (0.03 to 2.10)	0.04	3.67 (2.28 to 5.07)	<0.01	<0.01	8.40 (4.05 to 12.74)	<0.01	9.64 (6.34 to 12.94)	<0.01	0.65
Own education (26 years)	704/771	1.65 (0.69 to 2.60)	<0.01	2.08 (0.81 to 3.35)	<0.01	0.60	8.42 (4.43 to 12.41)	<0.01	3.41 (0.27 to 6.54)	0.03	0.05
Occupational class (53 years)	739/789	0.72 (−0.26 to 1.69)	0.15	2.14 (0.78 to 3.49)	<0.01	0.10	3.81 (−0.33 to 7.96)*	0.07	4.97 (1.69 to 8.25)	<0.01	0.67
Household income (60–64 years)	711/770	1.23 (0.32 to 2.14)*	<0.01	2.17 (0.91 to 3.42)*	<0.01	0.24	4.09 (0.16 to 8.01)	0.04	3.26 (0.20 to 6.32)	0.04	0.74

		(C) Appendicular lean mass index (kg/m^2^)									
	N (M/F)	Men, unadjusted	p Value	Men, adjusted for fat mass index	p Value	Women, unadjusted	p Value	Women, adjusted for fat mass index	p Value	p Value#	p Value##
Paternal occupational class (4 years)	710/767	0.31 (0.06 to 0.55)	0.01	0.17 (−0.04 to 0.39)	0.11	0.04 (−0.18 to 0.26)	0.70	−0.26 (−0.42 to −0.09)	<0.01	0.11	<0.01
Maternal education (6 years)	668/721	0.26 (−0.01 to 0.54)	0.06	0.10 (−0.14 to 0.34)	0.39	0.11 (−0.14 to 0.35)	0.40	−0.21 (−0.39 to −0.02)	0.03	0.40	0.03
Paternal education (6 years)	660/718	0.26 (−0.01 to 0.52)	0.06	0.12 (−0.11 to 0.36)	0.30	0.16 (−0.08 to 0.40)	0.20	−0.26 (−0.45 to −0.08)	<0.01	0.60	<0.01
Own education (26 years)	704/771	0.30 (0.05 to 0.55)*	0.02	0.09 (−0.13 to 0.31)*	0.42	−0.03 (−0.25 to 0.19)	0.82	−0.27 (−0.43 to −0.10)	<0.01	0.05	<0.01
Occupational class (53 years)	739/789	0.14 (−0.12 to 0.39)	0.30	0.04 (−0.18 to 0.27)	0.70	0.01 (−0.22 to 0.24)*	0.94	−0.24 (−0.41 to −0.06)	<0.01	0.47	0.04
Household income (60–64 years)	711/770	−0.08 (−0.32 to 0.16)	0.52	−0.24 (−0.45 to −0.03)	0.03	0.08 (−0.14 to 0.29)	0.47	−0.17 (−0.33 to −0.01)	0.04	0.34	0.73

#p Value for sex interaction term (#before and ##after adjustment for fat mass index); *evidence for departure from linearity (p<0.05); occupational class was that of the highest in the household and derived using the Registrar General's classification; analyses were restricted to those with valid measures for all body composition outcomes.

There was little evidence of deviations from linearity, except for associations of lower paternal and own occupational class (in men) and lower household income with higher fat mass and android to gynoid ratio, due to lower than expected fat mass levels in the lowest SEP group (see table 1).

### Associations between SEP and appendicular lean mass

In unadjusted analyses, lower SEP in childhood and lower own education were weakly associated with higher appendicular lean mass index in men, with no association in women (tables 1 and 2). These associations differed after adjustment for fat mass index, which was positively correlated with appendicular lean mass index (men=0.50, women=0.66): in men, lower household income was associated with lower lean mass index while other indicators of SEP were not associated; in women, lower levels of all indicators of SEP were associated with lower lean mass (table 2C). Compared with associations between SEP and fat mass, the magnitude of these was modest: for a given amount of fat mass among men, there was 0.24 (95%: −0.45 to −0.03) lower appendicular lean mass index (kg/m^2^) in the lowest compared with highest household income group, and 0.26 (95% CI −0.45 to −0.08) lower levels in the lowest compared with highest paternal education group among women.

### Are associations between childhood SEP and body composition explained by birth weight, childhood weight gain or adult SEP?

Paternal education, the indicator of childhood SEP most strongly associated with fat mass in women, was used in these analyses. Lower paternal education was weakly and non-significantly associated with lower birth weight (p=0.6 in men and p=0.2 in women), and was associated with lower weight gain from 0 to 7 years in men (p= 0.01) but not women (p=0.5), and with greater weight gain from 7 to 20 years (p<0.05 in both sexes).

Associations between lower paternal education and higher fat mass were not explained by birth weight or weight gain from 0 to 7, but were partly explained by weight gain from 7 to 20 years, by 22.6% in men and 15.2% in women (percentage change in SII; table 3). This association was largely explained by own education and adult SEP in men (74.2%) and partly explained in women (19.9%). Associations between lower paternal education and higher android to gynoid ratio were marginally explained by birth weight (2.2% in men and 0.9% in women) and weight gain from 0 to 7 (2.4% in men and 1.1% in women), and partly by weight gain from 7 to 20 years (7.4% in men and 7.0% in women). In both sexes, this association was partially but not wholly explained by indicators of adult SEP (34.7% in men and 8.0% in women).

**Table 3:** Differences in fat mass, android to gynoid ratio and lean mass (95% CI) at age 60–64 years between the hypothetical lowest and highest paternal educational attainment (slope index of inequality), with sequential adjustment for potential mediators

Men (n=479)	Fat mass index (kg/m^1.2^)	p Value	Android to gynoid fat mass ratio	p Value	Appendicular lean mass index (kg/m^2^), adjusted for fat mass index	p Value
1. Paternal education (6 years)	0.93 (−0.25 to 2.11)	0.12	8.50 (3.49 to 13.52)	<0.001		
2. Model 1+birth weight	0.93 (−0.25 to 2.12)	0.12	8.31 (3.34 to 13.28)	<0.001		
3. Model 1+weight gain from 0 to 7 years	1.13 (−0.04 to 2.29)	0.06	8.30 (3.31 to 13.29)	<0.001		
4. Model 1+weight gain from 7 to 20 years	0.72 (−0.39 to 1.82)	0.21	7.87 (2.83 to 12.92)	<0.001		
5. Model 1+own education and adult SEP	0.24 (−1.04 to 1.51)	0.72	5.55 (0.12 to 10.98)	0.05		
Women (n=515)						
1. Paternal education (6 years)	4.22 (2.62 to 5.82)	<0.001	10.62 (6.69 to 14.55)	<0.001	−0.27 (−0.49 to −0.06)	0.01
2. Model 1+birth weight	4.21 (2.61 to 5.82)	<0.001	10.52 (6.59 to 14.45)	<0.001	−0.26 (−0.48 to −0.05)	0.02
3. Model 1+weight gain from 0 to 7 years	4.24 (2.64 to 5.83)	<0.001	10.50 (6.57 to 14.42)	<0.001	−0.24 (−0.45 to −0.03)	0.03
4. Model 1+weight gain from 7 to 20 years	3.58 (2.06 to 5.10)	<0.001	9.88 (5.95 to 13.80)	<0.001	−0.26 (−0.47 to −0.05)	0.01
5. Model 1+own education and adult SEP	3.38 (1.54 to 5.23)	<0.001	9.77 (5.29 to 14.26)	<0.001	−0.12 (−0.36 to 0.13)	0.35

Occupational class was that of the highest in the household and derived using the Registrar General's classification; analyses were restricted to those with valid measures for each indicator of SEP, relevant potential mediators and all body composition outcomes. Cells are blank where analyses were not included due to lack of evidence for association in univariable analyses (p>0.05).

SEP, socioeconomic position.

The association between lower paternal education and lower appendicular lean mass index in women (after adjustment for fat mass) was partly explained by birth weight (3.7%) and weight gain from 0 to 7 (11.1%) and 7 to 20 years (3.7%). However, this association was largely explained by own educational attainment and adult SEP (55.6%).

The above associations were similar when maternal education or paternal occupational class was used (see online supplementary tables S1 and S2).

## Discussion

### Main findings

Lower SEP across life was associated with higher fat mass and android to gynoid ratio in early old age. Lower SEP across life was also associated with lower lean mass in women after adjustment for fat mass, but this association was only found with contemporaneous household income in men. Associations between lower childhood SEP and higher fat mass were partly explained by pubertal weight gain and adult SEP, whereas the association between lower childhood SEP and lower lean mass was largely explained by adult SEP.

### Comparison with previous studies

Findings from this study are consistent with previous NSHD studies which found lower childhood SEP and own education were associated with higher BMI at earlier ages and poorer physical performance.^3^ ^34–38^ We extend these by using a longer period of follow-up and direct measures of body composition.

This study has advantages compared with the few previous studies^11–19^ that reported inconsistent associations between SEP and direct measures of body composition by: using indicators of SEP across life; using android to gynoid ratio and appendicular lean mass; considering whether SEP was associated with lean mass independently of fat mass; and examining whether associations with childhood SEP were explained by preadulthood weight gain.

### Explanation of findings

The associations of SEP with fat and lean mass are likely to be explained by the socioeconomic patterning of the determinants of these masses which operate across life. Findings from this study suggest that childhood SEP differences in fat mass and fat distribution in early old age are partly attributable to socioeconomic differences in preadulthood weight gain. In this cohort, lower birth weight was associated with higher android to gynoid ratio, while greater weight gain from 7 to 20 years was associated with higher fat mass and higher android to gynoid ratio in late middle age, potentially due to the tracking of acquired fat mass and body fat distribution into adulthood.^26^ Associations with fat mass were not explained by prepubertal weight gain, although this could explain socioeconomic differences in fat mass in younger cohorts, where socioeconomic differences in fat accrual are likely to have manifest earlier.^39^

The association between lower childhood SEP and lower lean mass (after adjustment for fat mass) in women was largely explained by own education and adult SEP. This suggests that adulthood factors may largely explain this association, although SEP differences in muscle growth may not be fully captured by weight gain, which represent accrual of both muscle and fat.

Lower SEP was only associated with lower lean mass after adjustment for fat mass, and not before. While we hypothesised that this adjustment would reduce confounding due to fat mass, it is possible that it could have introduced bias due to overadjustment. Differences in unadjusted and confounder-adjusted associations do not inform us of which of these two scenarios is most likely. However, it should be noted that lean mass may be a more clinically meaningful outcome when adjustment is made for fat mass, as lean (muscle) mass is needed to support the weight of the body—as such, higher lean (muscle) mass without higher fat mass would be expected to lead to improved physical function and improved health. In support of this, lean mass has been found to be a stronger predictor of physical function after adjustment for fat mass.^6^ ^40^

Both childhood and adult SEP are also likely to influence fat and lean mass levels in later life by impacting on behavioural factors in adulthood. For example, lower childhood and adult SEP have been associated with lower leisure time physical activity participation,^41^ ^42^ which in turn may lead to higher fat and lower lean mass. While it is likely that SEP differences in diet partly explain findings, SEP has not been consistently associated with dietary factors thought to affect fat mass (total energy intake)^43^ and lean mass (protein intake);^44^ this may be due to inaccuracy in the self-reporting of diet intake which may vary by SEP and body weight.^43^ ^45^ SEP differences in physical activity and diet may in turn be due to financial and area-based differences in access to leisure activity and dietary resources.^43^ ^46^ These pathways are being investigated in the NSHD and warrant investigation in other cohorts.

Sex differences in associations are likely to reflect sex differences in the socioeconomic patterning of the determinants of fat and lean mass. In previous research on the NSHD, childhood SEP differences in adult BMI became stronger from 36 to 53 years in women, but remained stable in men.^37^ This suggests that the impact of childhood SEP on behaviours which lead to gains in fat mass may be stronger in women. Due to both biological (sex) and social/cultural (gender) differences, these determinants may differ in the extent to which they influence fat mass in men and women. For example, in the NSHD physical activity was more strongly inversely associated with fat mass in women than men.46a Women of lower SEP also experience additional risk factors for fat mass accrual which men do not (eg, childbirth), which could also contribute to sex differences in associations. Inconsistent associations between SEP and lean mass in men may be due to each SEP indicator capturing exposures with conflicting effects on male lean mass levels. For example, lower adult SEP is associated with less frequent leisure time physical activity participation,^41^ ^42^ but with more frequent weight-bearing occupational physical activity.^47^

Paternal education was more strongly associated with fat mass in women than other childhood SEP indicators, and additional analyses showed that only paternal education remained associated with fat mass when all childhood SEP indicators were mutually adjusted for (data available on request). In this cohort, paternal education may therefore have been more closely related to the factors that influence fat mass than either maternal education or paternal occupational class. In order to identify the extent to which this finding is contextual or generalisable and to elucidate underlying pathways, we need to examine whether the same patterns of association are found in other birth cohorts.

Associations between lower SEP and higher android to gynoid ratio were driven by the stronger associations between SEP and android than gynoid fat mass (shown in online supplementary table S3), and were not explained by adult height. While the determinants of fat distribution are not well understood, high levels of stress have been suggested as causing preferential deposition of abdominal fat,^48^ and physical activity may lead to greater losses of abdominal compared with peripheral fat.^49^ As such, SEP differences in stress and physical activity may partly explain these associations.

### Strengths and limitations

Strengths of this study include the use of multiple prospectively ascertained SEP indicators across life and direct measures of body composition. Regional measures were used which may be most relevant for health-related outcomes: appendicular lean mass excludes organ mass and is likely to be a more accurate measure of skeletal muscle mass than whole body lean mass, and abdominal fat distribution may influence health independently of whole body fat.^50^ ^51^ Unlike previous studies, fat and lean mass were adjusted for adult height and associations between SEP and lean mass were examined before and after adjustment for fat mass, an important potential confounder. Another strength is the availability of birth weight and preadulthood weight gain measures in the NSHD, potential mediators of associations between childhood SEP and adult body composition.

While more indicators of SEP were used than in previous studies, more refined measures of SEP may better capture SEP differences in body composition. For example, total household income is a relatively crude measure of disposable income as it does not account for household size, housing costs or debt payments. However, additional analyses showed similar results were obtained when household income was equivalised by household size, suggesting that this was unlikely to substantially affect the associations found.

As in all longitudinal studies, the NSHD has experienced attrition and this may have introduced bias. Previous analyses of this cohort have shown that on average participants with higher BMI and lower SEP (lower educational attainment and lower occupational class) at 53 years were less likely to attend the CRF at 60–64 years.^52^ This pattern of missing data is likely to have led to reduced power to detect the associations between lower SEP and higher fat mass. Among those with higher fat mass, it is possible that consequent loss to follow-up may have occurred more readily among those of lower SEP. For example, lower SEP has been associated with less favourable access to healthcare services,^53^ such that an adverse health or disability event caused in part by higher fat mass levels may be more likely to lead to loss to follow-up in obese participants of lower compared with higher SEP. This pattern of missing data would have led to an underestimation of the magnitude of SEP differences in fat mass in the present study. Additional analyses supported this, as SEP differences in BMI were greater when additionally including participants who attended home visits at 60–64 years (see online supplementary table S4 compared with online supplementary table S5).

There were some differences in unadjusted effect estimates when analyses were restricted to those with valid data for all SEP indicators and potential mediators. However, these differences did not result in changes to the conclusions of the results obtained, enabling the investigation of important mediating factors.

### Implications

Results suggest that reducing socioeconomic inequalities across life would have beneficial effects by leading to fewer individuals in later adulthood with higher fat mass, higher android to gynoid ratio and lower lean mass (for a given amount of fat mass). Reducing inequalities in childhood may be particularly beneficial by affecting both early life determinants of adult body composition and adult SEP.

Previous studies using BMI are likely to have underestimated SEP differences in fat mass if, as found in this study, lower SEP is consistently associated with higher fat but not higher lean mass. For example, when expressed as a percentage difference, the mean difference in fat mass index comparing the lowest with the highest paternal occupational class at 4 years (from SII) was 8.6% in men and 16.1% in women; as expected, the magnitude of these associations was weaker when BMI was used as the outcome—a 6.3% difference in BMI in men and 9% difference in women (see online supplementary table S4). Similarly, the magnitude of associations with waist to hip ratio was weaker than those using android to gynoid ratio (see online supplementary table S4).

The SEP differences in fat and lean mass found in this cohort may differ from those in younger and older birth cohorts. While participants from the 1958 British birth cohort study had higher BMI on average at 43–45 years than those in the NSHD (born 1946),^54^ further cross-cohort comparisons are required to examine cohort differences in the socioeconomic patterning of fat and lean mass, as well as the determinants of these socioeconomic differences.

## Conclusions

In both sexes, lower SEP across life was associated with higher fat mass and higher android to gynoid ratio in early old age. After adjustment for fat mass, lower SEP across life in women and lower contemporaneous household income in men were associated with lower lean mass. Associations between childhood SEP and body composition outcomes were partly explained by preadulthood weight gain and adult SEP.

**What is already known on this subject**

- In developed nations, lower socioeconomic position (SEP) in childhood and adulthood have been associated with higher adult body mass index. These associations are more consistent in women than men.
- Few studies have examined associations with direct measures of fat and lean mass which body mass index does not distinguish. Fat and lean mass are likely to have independent roles in health and physical functioning.

**What this study adds**

- In a British birth cohort study, lower SEP across life was associated with direct measures of higher fat mass and abdominal fat distribution in both sexes at 60–64 years. The relative SEP disparities in fat mass were larger when using direct measures of fat mass compared with anthropometric measures (body mass index and waist to hip ratio). After adjustment for fat mass, lower SEP across life was associated with lower lean mass in women but not men.
- Associations between childhood SEP and these outcomes were partly explained by preadulthood weight gain and adult SEP.
- Reducing socioeconomic inequalities across life, and particularly in childhood, may have beneficial effects by leading to fewer individuals in later adulthood with higher fat mass, higher abdominal fat distribution and lower lean mass (for a given amount of fat mass).

## Supplementary Material

Web tables

## References

[1] McLaren L Socioeconomic status and obesity. Epidemiol Rev 2007;29:29–48 17478442 10.1093/epirev/mxm001

[2] Senese LC Almeida ND Fath AK Associations between childhood socioeconomic position and adulthood obesity. Epidemiol Rev 2009;31:21–51 19648176 10.1093/epirev/mxp006 PMC2873329

[3] Strand B Cooper R Hardy R Lifelong socioeconomic position and physical performance in midlife: results from the British 1946 birth cohort. Eur J Epidemiol 2011;26:475–83 21416275 10.1007/s10654-011-9562-9 PMC3246593

[4] The Marmot Review Team. Fair Society, Healthy Lives: the strategic review of health inequalities in England, post 2010. The Marmot Review, 2010

[5] Wolfe RR The underappreciated role of muscle in health and disease. Am J Clin Nutr 2006;84:475–82 16960159 10.1093/ajcn/84.3.475

[6] Dufour AB Hannan MT Murabito JM Sarcopenia definitions considering body size and fat mass are associated with mobility limitations: the Framingham study. J Gerontol A Biol Sci Med Sci 2012;68:168–74 22503991 10.1093/gerona/gls109 PMC3598358

[7] Hla MM Davis JW Ross PD Multicenter study of the influence of fat and lean mass on bone mineral content: Evidence for differences in their relative influence at major fracture sites. Am J Clin Nutr 1996;64:354–60 8780345 10.1093/ajcn/64.3.345

[8] Srikanthan P Hevener AL Karlamangla AS Sarcopenia exacerbates obesity-associated insulin resistance and dysglycemia: findings from the National Health and Nutrition Examination Survey III. Plos One 2010;5:e10805 22421977 10.1371/journal.pone.0010805 PMC3279294

[9] Wang ZM Heshka S Zhang K Resting energy expenditure: systematic organization and critique of prediction methods. Obes Res 2001;9:331–6 11346676 10.1038/oby.2001.42

[10] Cruz-Jentoft AJ Baeyens JP Bauer JM Sarcopenia: European consensus on definition and diagnosis: Report of the European Working Group on Sarcopenia in Older People. Age Ageing 2010;39:412–23 20392703 10.1093/ageing/afq034 PMC2886201

[11] Yliharsila H Kajantie E Osmond C Body mass index during childhood and adult body composition in men and women aged 56–70 years. Am J Clin Nutr 2008;87:1769–75 18541567 10.1093/ajcn/87.6.1769

[12] Ekelund U Neovius M Linne Y Associations between physical activity and fat mass in adolescents: the Stockholm Weight Development Study. Am J Clin Nutr 2005;81:355–60 15699221 10.1093/ajcn.81.2.355

[13] Boot AM Bouquet J de Ridder MA Determinants of body composition measured by dual-energy X-ray absorptiometry in Dutch children and adolescents. Am J Clin Nutr 1997;66:232–8 9250099 10.1093/ajcn/66.2.232

[14] Gigante DP Victora CG Horta BL Undernutrition in early life and body composition of adolescent males from a birth cohort study. Br J Nutr 2007;97:949–54 17408527 10.1017/S0007114507433025

[15] Visser M Harris TB Langlois J Body fat and skeletal muscle mass in relation to physical disability in very old men and women of the Framingham Heart Study. J Gerontol A Biol Sci Med Sci 1998;53:M214–21 9597054 10.1093/gerona/53a.3.m214

[16] Lantz H Bratteby LE Fors H Body composition in a cohort of Swedish adolescents aged 15, 17 and 20.5 years. Acta Paediatr 2008;97:1691–7 18795912 10.1111/j.1651-2227.2008.01035.x

[17] Seppanen-Nuijten E Lahti-Koski M Mannisto S Fat free mass and obesity in relation to educational level. BMC Public Health 2009;9:448 19961589 10.1186/1471-2458-9-448 PMC2801678

[18] Al-Qaoud TM Nitsch D Wells J Socioeconomic status and reduced kidney Function in the Whitehall II study: role of obesity and metabolic syndrome. Am J Kidney Dis 2011;58:389–97 21719176 10.1053/j.ajkd.2011.04.017 PMC3192873

[19] Brennan SL Henry MJ Nicholson GC Socioeconomic status and risk factors for obesity and metabolic disorders in a population-based sample of adult females. Prev Med 2009;49:165–71 19576925 10.1016/j.ypmed.2009.06.021

[20] Forbes GB Some adventures in body composition, with special reference to nutrition. Acta Diabetol 2003;40:s238–41 14618482 10.1007/s00592-003-0075-1

[21] Chaston TB Dixon JB O'Brien PE Changes in fat-free mass during significant weight loss: a systematic review. Int J Obes 2006;31:743–50 10.1038/sj.ijo.0803483 17075583

[22] Wells JCK Marphatia AA Cole TJ Associations of economic and gender inequality with global obesity prevalence: Understanding the female excess. Soc Sci Med 2012;75:482–90 22580078 10.1016/j.socscimed.2012.03.029

[23] Hoyenga KB Hoyenga KT Gender and energy balance: sex differences in adaptations for feast and famine. Physiol Behav 1982;28:545–63 7043508 10.1016/0031-9384(82)90153-6

[24] Silva LM Jansen PW Steegers EA Mother's educational level and fetal growth: the genesis of health inequalities. Int J Epidemiol 2010;39:1250–61 20478844 10.1093/ije/dyq069

[25] Yliharsila H Kajantie E Osmond C Birth size, adult body composition and muscle strength in later life. Int J Obes 2007;31:1392–9 10.1038/sj.ijo.0803612 17356523

[26] Bann D Wills AK Cooper R Birth weight and growth from infancy to late adolescence in relation to fat and lean mass in early old age: findings from the MRC National Survey of Health and Development. Int J Obes 2014;38:69–75 10.1038/ijo.2013.115 PMC3884138 23779050

[27] Kuh D Hardy R Butterworth S Developmental origins of midlife physical performance: evidence from a british birth cohort. Am J Epidemiol 2006;164:110–21 16757569 10.1093/aje/kwj193

[28] Wadsworth ME Follow-up of the first national birth cohort: findings from the Medical Research Council National Survey of Health and Development. Paediatr Perinat Epidemiol 1987;1:95–117 3333433 10.1111/j.1365-3016.1987.tb00093.x

[29] Kuh D Pierce M Adams J Updating the cohort profile for the MRC National Survey of Health and Development: a new clinic-based data collection for ageing research. Int J Epidemiol 2011;40:e1–9 21345808 10.1093/ije/dyq231 PMC3043283

[30] Plank LD Dual-energy X-ray absorptiometry and body composition. Curr Opin Clin Nutr Metab Care 2005;8:305–9 15809534 10.1097/01.mco.0000165010.31826.3d

[31] Department of Education and Science. Burnham Further Education Committee grading courses. London, UK: HMSO, 1972

[32] Wells JCK Cole TJ; ALSPAC study team. Adjustment of fat-free mass and fat mass for height in children aged 8 years. Int J Obes 2002;26:947–52 10.1038/sj.ijo.0802027 12080448

[33] Mackenbach JP Kunst AE Measuring the magnitude of socio-economic inequalities in health: an overview of available measures illustrated with two examples from Europe. Soc Sci Med 1997;44:757–71 9080560 10.1016/s0277-9536(96)00073-1

[34] Langenberg C Hardy R Kuh D Central and total obesity in middle aged men and women in relation to lifetime socioeconomic status: evidence from a national birth cohort. J Epidemiol Community Health 2003;57:816–22 14573589 10.1136/jech.57.10.816 PMC1732299

[35] Hardy R Wadsworth M Kuh D The influence of childhood weight and socioeconomic status on change in adult body mass index in a British national birth cohort. Int J Obes 2000;24:725–34 10.1038/sj.ijo.0801238 10878679

[36] Murray ET Mishra GD Kuh D Life Course models of socioeconomic position and cardiovascular risk factors: 1946 birth cohort. Ann Epidemiol 2011;21:589–97 21737047 10.1016/j.annepidem.2011.04.005 PMC3226834

[37] Strand B Murray ET Guralnik J Childhood social class and adult adiposity and blood-pressure trajectories 36–53 years: gender-specific results from a British birth cohort. J Epidemiol Community Health 2012;66:512–18 21098826 10.1136/jech.2010.115220 PMC3491863

[38] Hurst L Stafford M Cooper R Lifetime socioeconomic inequalities in physical and cognitive ageing. Am J Public Health 2013;103:1641–8 23865666 10.2105/AJPH.2013.301240 PMC3780680

[39] Howe L Tilling K Galobardes B Socioeconomic disparities in trajectories of adiposity across childhood. Int J Pediatr Obes 2011;6 (2 Part 2):e144–53 20860432 10.3109/17477166.2010.500387 PMC5102325

[40] Newman AB Kupelian V Visser M Sarcopenia: alternative definitions and associations with lower extremity function. J Am Geriatr Soc 2003; 51:1602–9 14687390 10.1046/j.1532-5415.2003.51534.x

[41] Silverwood RJ Pierce M Nitsch D Is Intergenerational social mobility related to the type and amount of physical activity in mid-adulthood? Results from the 1946 British Birth Cohort Study. Ann Epidemiol 2012;22:487–98 22534178 10.1016/j.annepidem.2012.03.002 PMC3383988

[42] Kuh DJ Cooper C Physical activity at 36 years: patterns and childhood predictors in a longitudinal study. J Epidemiol Community Health 1992;46:114–19 1583424 10.1136/jech.46.2.114 PMC1059517

[43] Giskes K Avendano M Rug J A systematic review of studies on socioeconomic inequalities in dietary intakes associated with weight gain and overweight/obesity conducted among European adults. Obesity Reviews 2010;11:413–29 19889178 10.1111/j.1467-789X.2009.00658.x

[44] Darmon N Drewnowski A Does social class predict diet quality? Am J Clin Nutr 2008;87:1107–17 18469226 10.1093/ajcn/87.5.1107

[45] Lissner L Troiano RP Midthune D OPEN about obesity: recovery biomarkers, dietary reporting errors and BMI. Int J Obes 2007;31:956–61 10.1038/sj.ijo.0803527 17299385

[46] Stafford M Cummins S Ellaway A Pathways to obesity: Identifying local, modifiable determinants of physical activity and diet. Soc Sci Med 2007;65:1882–97 17640787 10.1016/j.socscimed.2007.05.042

[46a] Bann D, Kuh D, Wills AK, *et al*. Physical activity across adulthood in relation to fat and lean mass in early old age: findings from the MRC National Survey of Health and Development, 1946–2010. *Am J Epidemiol*. In press 10.1093/aje/kwu033 PMC4010186 24722997

[47] Beenackers M Kamphuis C Giskes K Socioeconomic inequalities in occupational, leisure-time, and transport related physical activity among European adults: a systematic review. Int J Behav Nutr Phys Act 2012;9:116 22992350 10.1186/1479-5868-9-116 PMC3491027

[48] Björntorp P Do stress reactions cause abdominal obesity and comorbidities? Obes Rev 2001;2:73–86 12119665 10.1046/j.1467-789x.2001.00027.x

[49] Ohkawara K Tanaka S Miyachi M A dose-response relation between aerobic exercise and visceral fat reduction: systematic review of clinical trials. Int J Obes 2007;31:1786–97 10.1038/sj.ijo.0803683 17637702

[50] Wajchenberg BL Subcutaneous and visceral adipose tissue: their relation to the metabolic syndrome. Endocr Rev 2000;21:697–738 11133069 10.1210/edrv.21.6.0415

[51] Wiklund P Toss F Jansson JH Abdominal and gynoid adipose distribution and incident myocardial infarction in women and men. Int J Obes 2010;34: 1752–8 10.1038/ijo.2010.102 20498655

[52] Stafford M Black S Shah I Using a birth cohort to study ageing: representativeness and response rates in the National Survey of Health and Development. Eur J Ageing 2013;10:145–57 23637643 10.1007/s10433-013-0258-8 PMC3637651

[53] Dixon A Le GJ Henderson J Is the British National Health Service equitable? The evidence on socioeconomic differences in utilization. J Health Serv Res Policy 2007;12:104–9 17407661 10.1258/135581907780279549

[54] Li L Hardy R Kuh D Child-to-adult body mass index and height trajectories: a comparison of 2 British Birth Cohorts. Am J Epidemiol 2008;168:1008–15 18801885 10.1093/aje/kwn227 PMC3159394

