# Supplementary material for: Socioeconomic position across life and body composition in early old age: findings from a British birth cohort study
Source: J Epidemiol Community Health. 2014 Feb 24;68(6):516–23. doi: 10.1136/jech-2013-203373 (PMC4033171; doi:10.1136/jech-2013-203373)
Supplement: Web tables [file jech-2013-203373-s1.pdf]

Supplementary Table 1. Differences in fat mass, android: gynoid ratio, and lean mass (95% CI) at age 60–64 years between the hypothetical lowest and highest maternal educational attainment (slope index of inequality), with sequential adjustment for potential mediators

Men (n=486)	Fat mass index (kg/m$^{1.2}$)	P	Android: gynoid fat mass ratio	P	Appendicular lean mass index (kg/m^{2}), adjusted for fat mass index	P
1. Maternal education (6y)	1.32 (0.13, 2.50)	0.03	9.14 (4.11, 14.18)	<0.001		
2. Model 1 + birth weight	1.32 (0.13, 2.51)	0.03	9.34 (4.36, 14.32)	<0.001		
3. Model 1 + weight gain from 0-7y	1.69 (0.51, 2.88)	0.01	9.34 (4.30, 14.39)	<0.001		
4. Model 1 + weight gain from 7-20y	1.17 (0.05, 2.30)	0.04	8.23 (3.14, 13.31)	<0.001		
5. Model 1 + own education & adult SEP	0.67 (-0.61, 1.95)	0.31	6.17 (0.74, 11.60)	0.03		
**Women (n=517)**						
1. Maternal education (6y)	3.12 (1.45, 4.79)	<0.001	6.41 (2.31, 10.52)	<0.001	-0.19 (-0.41, 0.03)	0.09
2. Model 2 + birth weight	3.13 (1.45, 4.81)	<0.001	6.15 (2.02, 10.27)	<0.001	-0.16 (-0.38, 0.06)	0.16
3. Model 1 + weight gain from 0-7y	3.14 (1.48, 4.81)	<0.001	6.13 (2.01, 10.26)	<0.001	-0.14 (-0.36, 0.07)	0.19
4. Model 1 + weight gain from 7-20y	2.68 (1.10, 4.25)	<0.001	5.79 (1.70, 9.87)	0.01	-0.17 (-0.38, 0.04)	0.12
5. Model 1 + own education & adult SEP	2.20 (0.37, 4.04)	0.02	4.67 (0.19, 9.15)	0.04	-0.06 (-0.30, 0.19)	0.65

Notes: occupational class was that of the highest in the household and derived using the Registrar General's classification; analyses were restricted to those with valid measures for each indicator of socioeconomic position, relevant potential mediators and all body composition outcomes. Cells are blank where analyses were not included due to lack of evidence for association in univariable analyses (p>0.05)

Supplementary Table 2. Differences in fat mass, android: gynoid ratio, and lean mass (95% CI) at age 60–64 years between the hypothetical lowest and highest paternal occupational class (slope index of inequality), with sequential adjustment for potential mediators

Men (n=501)	Fat mass index (kg/m$^{1.2}$)	P	Android: gynoid fat mass ratio	P	Appendicular lean mass index (kg/m^2), adjusted for fat mass index	P
1. Paternal occupational class (4y)	1.02 (-0.10, 2.15)	0.07	7.93 (3.13, 12.72)	<0.001		
2. Model 1 + birth weight	1.03 (-0.10, 2.15)	0.07	8.06 (3.30, 12.81)	<0.001		
3. Model 1 + weight gain from 0-7y	1.40 (0.28, 2.52)	0.01	8.17 (3.35, 12.99)	<0.001		
4. Model 1 + weight gain from 7-20y	0.87 (-0.20, 1.94)	0.11	7.17 (2.30, 12.04)	<0.001		
5. Model 1 + own education & adult SEP	0.27 (-0.97, 1.50)	0.67	4.53 (-0.74, 9.80)	0.09		
**Women (n=533)**						
1. Paternal occupational class (4y)	3.13 (1.62, 4.65)	<0.001	6.80 (3.10, 10.51)	<0.001	-0.24 (-0.44, -0.04)	0.02
2. Model 1 + birth weight	3.14 (1.62, 4.66)	<0.001	6.68 (2.97, 10.39)	<0.001	-0.23 (-0.42, -0.03)	0.03
3. Model 1 + weight gain from 0-7y	3.35 (1.85, 4.86)	<0.001	6.61 (2.89, 10.33)	<0.001	-0.17 (-0.36, 0.03)	0.09
4. Model 1 + weight gain from 7-20y	2.95 (1.52, 4.37)	<0.001	6.18 (2.49, 9.87)	<0.001	-0.18 (-0.37, 0.01)	0.07
5. Model 1 + own education & adult SEP	2.27 (0.58, 3.96)	0.01	5.25 (1.14, 9.36)	0.01	-0.09 (-0.31, 0.13)	0.41

Notes: occupational class was that of the highest in the household and derived using the Registrar General's classification; analyses were restricted to those with valid measures for each indicator of socioeconomic position, relevant potential mediators and all body composition outcomes. Cells are blank where analyses were not included due to lack of evidence for association in univariable analyses (p>0.05)

Supplementary Table 3. Differences in android and gynoid fat mass (95% CI) at age 60–64 years between the hypothetical lowest and highest socioeconomic position (slope index of inequality)

		Android fat mass (kg)				Gynoid fat mass (kg)			
	N (M/F)	Men	P	Women	P	Men	P	Women	P
Paternal occ. class (4y)	710/767	0.27(0.02, 0.52)	0.03	0.48(0.23, 0.73)	<0.01	-0.02(-0.28, 0.24)	0.89	0.48(0.11, 0.84)	0.01
Maternal education (6y)	668/721	0.34(0.06, 0.62)	0.02	0.55(0.27, 0.82)	<0.01	0.09(-0.21, 0.39)	0.54	0.61(0.20, 1.01)	<0.01
Paternal education (6y)	660/718	0.34(0.06, 0.62)	0.02	0.75(0.48, 1.02)	<0.01	0.03(-0.27, 0.32)	0.85	0.68(0.29, 1.08)	<0.01
Own education (26y)	704/771	0.42(0.19, 0.65)	<0.01	0.37(0.10, 0.64)	<0.01	0.17(-0.08, 0.43)	0.17	0.41(0.02, 0.81)	0.04
Occupational class (53y)	739/789	0.18(-0.07, 0.43)	0.16	0.46(0.19, 0.73)	<0.01	0.02(-0.24, 0.29)	0.86	0.48(0.08, 0.88)	0.02
Household income (60-64y)	711/770	0.30(0.07, 0.54)	0.01	0.42(0.17, 0.67)	<0.01	0.20(-0.05, 0.46)	0.12	0.52(0.15, 0.89)	<0.01

Notes: Occupational class was that of the highest in the household and derived using the Registrar General's classification; analyses were restricted to those with valid measures for all body composition outcomes.

Supplementary Table 4. Differences in anthropometric indicators of fat mass (95% CI) at age 60–64 years between the hypothetical lowest and highest socioeconomic position (slope index of inequality)—analyses restricted to those with valid measures for all DXA body composition outcomes

	N (M/F)	Body mass index (kg/m^2)					Waist circumference (cm)				
		Men	P	Women	P	P#	Men	P	Women	P	P#
Paternal occ. class (4y)	710/767	1.75(0.72, 2.78)	<0.01	2.47(1.22, 3.72)	<0.01	0.39	2.96(0.19, 5.73)	0.04	5.34(2.26, 8.42)	<0.01	0.26
Maternal education (6y)	668/721	1.83(0.69, 2.97)	<0.01	2.50(1.08, 3.91)	<0.01	0.48	4.09(1.02, 7.16)	<0.01	5.23(1.78, 8.68)	<0.01	0.63
Paternal education (6y)	660/718	1.57(0.45, 2.69)	<0.01	3.43(2.06, 4.81)	<0.01	0.04	3.95(0.94, 6.97)	0.01	7.53(4.17, 10.89)	<0.01	0.12
Own education (26y)	704/771	2.27(1.24, 3.31)	<0.01	1.82(0.57, 3.07)	<0.01	0.59	6.06(3.27, 8.84)	<0.01	4.31(1.20, 7.42)	<0.01	0.42
Occupational class (53y)	739/789	1.11(0.05, 2.17)	0.04	1.86(0.52, 3.19)	<0.01	0.39	2.95(0.10, 5.81)	0.04	4.79(1.52, 8.06)	<0.01	0.41
Household income (60-64y)	711/770	0.88(-0.12, 1.87)	0.09	2.01(0.77, 3.25)	<0.01	0.17	2.96(0.25, 5.67)	0.03	4.59(1.55, 7.63)	<0.01	0.44

	N (M/F)	Hip circumference (cm)					Waist: hip ratio				
		Men	P	Women	P	P#	Men	P	Women	P	P#
Paternal occ. class (4y)	710/767	-0.06(-1.94, 1.82)	0.95	3.09(0.47, 5.71)	0.02	0.06	0.03(0.01, 0.05)	<0.01	0.03(0.01, 0.04)	<0.01	0.81
Maternal education (6y)	668/721	1.38(-0.71, 3.47)	0.20	4.30(1.35, 7.25)	<0.01	0.12	0.03(0.01, 0.05)	<0.01	0.01(0.00, 0.03)	0.14	0.32
Paternal education (6y)	660/718	0.53(-1.53, 2.59)	0.61	5.88(3.00, 8.76)	<0.01	<0.01	0.03(0.01, 0.05)	<0.01	0.02(0.01, 0.04)	<0.01	0.51
Own education (26y)	704/771	1.22(-0.69, 3.12)	0.21	2.67(0.05, 5.29)	0.05	0.39	0.05(0.03, 0.06)	<0.01	0.02(0.00, 0.04)	0.02	0.02
Occupational class (53y)	739/789	0.28(-1.65, 2.22)	0.77	3.14(0.36, 5.91)	0.03	0.10	0.03(0.01, 0.04)	<0.01	0.02(0.00, 0.04)	0.02	0.68
Household income (60-64y)	711/770	-0.08(-1.92, 1.76)	0.93	3.78(1.21, 6.35)	<0.01	0.02	0.03(0.01, 0.04)	<0.01	0.01(0.00, 0.03)	0.09	0.21

Note: mean (SD) are as follows:
Body mass index (kg/m^2): 27.74 (3.94) in men, 27.51 (5.02) in women
Waist circumference (cm): 100.27 (10.56) in men, 91.47 (12.35) in women
Hip circumference (cm): 104.14 (7.14) in men, 105.90 (10.44) in women
Waist-hip ratio: 0.96 (0.06) in men, 0.86 (0.07) in women.

Occupational class was that of the highest in the household and derived using the Registrar General's classification

Supplementary Table 5. Differences in anthropometric indicators of fat mass (95% CI) at age 60–64 years between the hypothetical lowest and highest socioeconomic position (slope index of inequality)—analyses restricted to those with valid measures for anthropometric outcomes

		Body mass index (kg/m^2)					Waist circumference (cm)				
	N (M/F)	Men	P	Women	P	P#	Men	P	Women	P	P#
Paternal occ. class (4y)	1013/1095	1.92(1.03, 2.81)	<0.01	3.32(2.17, 4.47)	<0.01	0.06	4.01(1.61, 6.41)	<0.01	6.94(4.22, 9.66)	<0.01	0.12
Maternal education (6y)	955/1029	1.97(0.97, 2.97)	<0.01	3.65(2.31, 4.98)	<0.01	0.05	4.62(1.92, 7.33)	<0.01	7.48(4.35, 10.61)	<0.01	0.18
Paternal education (6y)	944/1023	1.88(0.91, 2.86)	<0.01	3.94(2.64, 5.24)	<0.01	0.01	5.19(2.56, 7.83)	<0.01	8.39(5.34, 11.44)	<0.01	0.12
Own education (26y)	1003/1100	2.44(1.54, 3.34)	<0.01	2.74(1.58, 3.90)	<0.01	0.69	6.89(4.48, 9.30)	<0.01	6.35(3.59, 9.10)	<0.01	0.77
Occupational class (53y)	1053/1119	1.48(0.57, 2.39)	<0.01	1.98(0.83, 3.14)	<0.01	0.51	4.44(2.00, 6.88)	<0.01	4.08(1.24, 6.91)	<0.01	0.85
Household income (60-64y)	1004/1086	1.17(0.29, 2.05)	<0.01	2.41(1.27, 3.54)	<0.01	0.09	3.39(1.01, 5.76)	<0.01	5.13(2.45, 7.81)	<0.01	0.34

		Hip circumference (cm)					Waist: hip ratio				
	N (M/F)	Men	P	Women	P	P#	Men	P	Women	P	P#
Paternal occ. class (4y)	1013/1095	1.04(-0.59, 2.67)	0.21	4.81(2.43, 7.18)	<0.01	0.01	0.03(0.02, 0.04)	<0.01	0.03(0.01, 0.04)	<0.01	0.78
Maternal education (6y)	955/1029	1.59(-0.25, 3.43)	0.09	6.24(3.49, 8.98)	<0.01	<0.01	0.03(0.02, 0.05)	<0.01	0.02(0.00, 0.04)	0.02	0.29
Paternal education (6y)	944/1023	1.30(-0.50, 3.10)	0.16	6.42(3.74, 9.10)	<0.01	<0.01	0.04(0.02, 0.05)	<0.01	0.03(0.01, 0.04)	<0.01	0.35
Own education (26y)	1003/1100	1.58(-0.08, 3.24)	0.06	4.26(1.87, 6.65)	<0.01	0.08	0.05(0.04, 0.06)	<0.01	0.02(0.01, 0.04)	<0.01	<0.01
Occupational class (53y)	1053/1119	0.78(-0.88, 2.44)	0.36	3.07(0.64, 5.50)	0.01	0.13	0.03(0.02, 0.05)	<0.01	0.01(0.00, 0.03)	0.10	0.04
Household income (60-64y)	1004/1086	0.37(-1.24, 1.99)	0.65	4.15(1.84, 6.46)	<0.01	0.01	0.03(0.02, 0.04)	<0.01	0.02(0.00, 0.03)	0.03	0.16

Notes: Occupational class was that of the highest in the household and derived using the Registrar General’s classification
